# New efficient meta-fermentation process for lactic acid production from municipal solid waste

**DOI:** 10.1186/s12934-022-01960-9

**Published:** 2022-11-05

**Authors:** Miguel G. Acedos, Paz Gómez-Pérez, Tamara Espinosa, Christian Abarca, Bernat Ibañez, Begoña Ruiz

**Affiliations:** 1 Biotechnology Department, AINIA, Parque Tecnológico de Valencia, Paterna, Spain; 2 Reciclados Palancia-Belcaire S.L., Algimia de Alfara, Spain

**Keywords:** Lactic acid, Meta-fermentation, Anaerobic Digestion, Bioaugmentation, OFMSW, Scale-up

## Abstract

**Background:**

The global market for lactic acid is witnessing growth on the back of increasing applications of lactic acid for manufacturing polylactic acid. Indeed, the lactic acid market is expected to reach 9.8 billion US dollars by 2025. The new concept of meta-fermentation has been proposed in recent years as an alternative to fermentation with pure cultures, due to multiple advantages such as lower susceptibility to contamination, no need for sterilization of culture media or lower raw material costs. However, there are still challenges to overcome to increase the conversion efficiency, decrease formation of by-products and facilitate fermentation control. In this context, the purpose of the study was to develop a robust meta-fermentation process to efficiently produce lactic acid from the OFMSW, stable at pre-industrial scale (1500 L). To maximize lactic acid production, operating conditions (pH, HRT) were modified, and a novel bioaugmentation strategy was tested.

**Results:**

A LAB-rich inoculum was generated with LAB isolated from the digestate and grown in the laboratory with MRS medium. After feeding this inoculum to the digester (bioaugmentation), lactic acid accumulation up to 41.5 gO_2_/L was achieved under optimal operating conditions. This corresponds to more than 70% of the filtered COD measured in the digestate. The amount of lactic acid produced was higher than the volatile fatty acids under all feeding strategies applied.

**Conclusions:**

The operating conditions that enhanced the production of lactic acid from mixed cultures were 55ºC, 2 days HRT and pH 4.8–5.7, with pH-control once a day. The bioaugmentation strategy improved the results obtained in the prototype without applying reinoculation. Lactic acid was the main product along with other carboxylic acids. Further improvements are needed to increase purity as well as lactic acid concentration to reach economic feasibility of the whole process (digestion of OFMSW and downstream).

**Supplementary Information:**

The online version contains supplementary material available at 10.1186/s12934-022-01960-9.

## Background

There is a growing interest in developing new bioprocesses for the treatment of municipal waste that are more efficient and environmentally friendly, allowing the products and waste generated in these activities to be valorised. Treatment options for the organic fraction of municipal solid waste (OFMSW) include landfilling, composting or anaerobic digestion for biogas production. In recent years, alternative technologies have been developed to produce biobased chemicals from OFMSW, such as through the carboxylate or volatile fatty acids (VFAs) platforms [1–3].

Fermentation processes have attracted attention for the sustainable production of value-added compounds such as biofuels and other bio-based chemical compounds from renewable sources [\*MERGEFORMAT 4]. Among them, the production of optically pure lactic acid (LA) isomer (D-/L-LA) and butanol has been reported from multiple renewable resources such as starch, lignocellulosic biomasses, or food waste in various fermentation processes with pure cultures [5–11]. Lactic acid is an important biobased chemical platform necessary to produce PLA bioplastic that is currently mainly produced by microbial fermentation from pure food carbohydrate cultures [11].

The global market for lactic acid (LA) is witnessing growth on the back of increasing applications of lactic acid for manufacturing of PLA. In addition, rising demand from other end user industries such as personal care products and food and beverages are other factors contributing towards the market growth. In 2014, food and beverages applications accounted for the largest share of the market pie (more than 40%) followed by industrial applications. However, industrial applications are projected to be the fastest growth application of lactic acid. It is projected to grow at a CAGR of 18.3% from 2015 to 2022 [12].

Lactic acid is usually produced using pure cultures and pure fermentable sugars [13–15]. A concept of meta-fermentation to produce LA has been proposed [5] consisting of the fermentative production of bio-based chemicals and fuels by a controlled mixed culture. Meta-fermentation has multiple advantages such as the possibility of using complex substrates, no sterile conditions requiredand lower operational and raw material costs.

The use of OFMSW as a fermentation substrate in a mixed culture system could reduce the current costs of LA production about 35–38% [16–18], reduce the total amount of waste generated, and save resources, thus, providing an environmental sustainable and economically efficient waste treatment procedure. Previous studies show that OFMSW can be a viable substrate for LA production [19], increasing and diversifying at the same time the possibilities of OFMSW management since it is possible to obtain a valuable bioproduct (LA),other bioproducts (*Lactobacili spp.* from digestate to be used as organic biostimulant) and fermented material for bioenergy production such us biogas.

The Table 1 shows a summary of the results obtained in previous works to produce lactic acid using mixed cultures and OFMSW as substrate. As it can be seen, up to 28.4 g_COD_/L of lactic acid using OFMSW and working in batch reactors were achieved [17].

**Table 1:** Results of lactic acid production from previous studies using mixed cultures

Substrate	Type of operation	Temperature (ºC)	pH (-)	HRT (d)	OLR (g_COD_/L·d)	Lactic acid (g_COD_/L)	Refs.
Glucose	Continuous	55	5	0.5	50	23	[24]
50% cooked rice + dry dog food	Semi-continuous	35	Not controlled	15	5 g_TS_/L·d	16	[22]
Food waste + sewage sludge	Batch	35	9	NA	NA	25.5	[25]
	Batch	50	7	NA	NA	21	
Food waste	Batch	37	5	NA	NA	20.7–28.4	[17]

*NA* Not Applicable, *HRT* hydraulic retention time, *OLR* organic loading rate

However, purification and obtaining an optically pure product from OFMSW are issues that remain to be solved [20]. Novel purification methods with lower environmental impact have been proposed for obtaining LA from fermentation broths, bringing this problem closer to resolution [21].

A strategy to produce optically pure L-LA based on modifying the operating and control parameters of the meta-fermentation using a mixed culture is therefore proposed in previous studies. Specifically, mixed pH control strategies are proposed for the fermentative production under thermophilic conditions of optically pure L-LA in a mixed culture system from OFMSW. The results obtained in recent studies present a pH control strategy successfully applied for the efficient production of optically pure meta-fermented LA based on a different concept from that employed in pure culture systems [9, 22]. In meta-fermentations under thermophilic conditions with oscillating pH control and switching to constant pH, optical purity of the L-LA isomer was achieved using unsterilised kitchen waste with preferential growth of the heterofermentative microorganism *Bacillus coagulans* (L-LA producer). Abdel-Rahman MA and colleagues [14] proposed to look for a suitable inoculum (bioaugmentation) as a seed for the meta-fermentation process, for which different microbial consortia from different composts or from mixed cultures developed in the laboratory. This strategy is already included in some published studies on the production of LA with mixed cultures [9, 23].

Bioaugmentation strategy is usually aimed at increasing biogas production in the case of single-stage anaerobic digestion, or to maximise VFAs synthesis and biogas production in two-stage digestions [26, 27]. Other authors have focused on bioaugmentation techniques to maximise hydrogen production in anaerobic digesters by using pure culture inoculum of different species [28, 29], in these cases bioaugmentation is aimed at increasing the production of LA that will subsequently be transformed to hydrogen by another bacteria group. In the case of bioaugmentation of digesters to increase the production of LA as the final product of digestion, the references found are more recent [23, 30]. These strategies are based on the use of inoculum with pure strains of lactic acid bacteria (LAB) and studying the effect under different operating conditions.

In this study, the aim is to use the digestate produced in the digester as a source of LAB, that once isolated with selective culture media, will be used as inoculum for bioaugmentation.

With this strategy, we aimed to improve the lactic acid production from OFMSW using mixed cultures. For this purpose, studies have been carried out in lab and pilot scale digester.

## Results

### Substrate characterization

Table 2 summarizes the results of the characterization of OFMSW samples used for the experiments at lab and pilot scale.

**Table 2:** Results of the characterisation of the diluted OFMSW samples used as substrate (mean value ± standard deviation)

Sample	TS (%)	VS (%TS)	C (g/100 g)	N (g/100 g)	C/N (-)
Substrate of trials in 36L-digester	12.5 ± 2.5	86.4 ± 4.3	NA	NA	NA
Substrate of trials in 1500L-digester	7.6 ± 1.9	88.7 ± 3.5	4.4 ± 1.1	0.2 ± 0.1	22.3 ± 4.2

*NA* not analysed, *TS* total solid, *VS* volatile solids, *C* carbon, *N* nitrogen

As it can be seen, total solids of diluted OFMSW for lab and pilot trials were below 15%, having on average a lower total solids content in the pilot tests than in lab tests which is associated with variability of OFMSW before dilution, while the values of volatile solids were very similar in both tests, observing an ash content below 20%.

### Lab-scale experiments: optimisation of pH and HRT

Table 3 shows the average results for each feeding strategy at lab scale.

**Table 3:** Summary of operating parameters and average results of lactic acid production in OFMSW fermentation trials at lab scale

Nr. of Feeding strategy	Duration (d)	Operational conditions		Results 36-L digester								
		OLR (kg_VS_/m^3^·d)	HRT (d)	pH (-)	TS (%)		Lactic acid (g/100 g)	Lactic acid (g/gTS)	Lactic acid (gCOD/kg)	Lactic acid (gCOD/L)^c^	% D-Lactic	% L-Lactic
1	19	4	8	4.58 ± 0.15	10.30 ± 3.75		1.26 ± 0.11	0.14 ± 0.06	14.18 ± 0.45	14.75	–	–
2	12	5	6	4.65 ± 0.17	5.20 ± 0.28		0.94 ± 0.38	0.18 ± 0.06	10.03 ± 4.07	10.43	–	–
3	18	13	4	4.40 ± 0.10	9.17 ± 3.66		0.43 ± 0.10	0.05 ± 0.02	4.59 ± 1.07	4.77	–	–
4	18	15	3	4.65 ± 0.21	6.40 ± 1.70		0.81 ± 0.15	0.13 ± 0.06	8.59 ± 1.58	8.93	53	47
5	21	24	2	4.68 ± 0.12	8.10 ± 0.69		1.24 ± 0.06	0.15 ± 0.01	13.26 ± 0.65	13.79	50 ± 1	50 ± 1
6	9	37	2^a^	4.73 ± 0.27	7.65 ± 0.64		1.33 ± 0.20	0.15 ± 0.04	14.19 ± 2.11	14.75	55 ± 6	45 ± 6
7	9	28	2^b^	5.03 ± 0.05	8.90		1.46	0.16	15.57	16.20	49	51
8	21	32	2^a^	4.75 ± 0.29	6.83 ± 1.01		1.56 ± 0.54	0.24 ± 0.10	15.92 ± 4.98	16.56	53 ± 3	47 ± 3

*HRT* hydraulic retention time, *OLR* organic loading rate, *TS* total solids

^a^pH-Adjustment at 7, once a day

^b^Without pH-Adjustment

^c^Average value transformed considering the digestate density (1.04)

The pH value remained naturally between 4.4–4.7 without pH control in the feeding strategy Nr.1–5.

From feeding strategy Nr.6 onwards, pH in the digester was artificially raised above 5, to approach the optimal pH for the growth of many LAB (5.5–6) [11], resulting in obtaining an average pH value of 4.7–5.0 in feeding strategies Nr.6–8.

At feeding strategies Nr.1–5,the maximum LA production was obtained at the lower HRT tested (2 days). At this point, the pH adjustment strategies were applied, observing a higher LA yield in the feeding strategies with a pH in the media close to 5. The percentage of D-/L isomers in this feeding strategy of the lab scale trials, was 53 and 47% respectively. The operational conditions of the last feeding strategy yielded the best results in terms of lactic acid production and quality. Thus, they were taken as the basis for the pilot scale experiments.

**Fig. 1:**
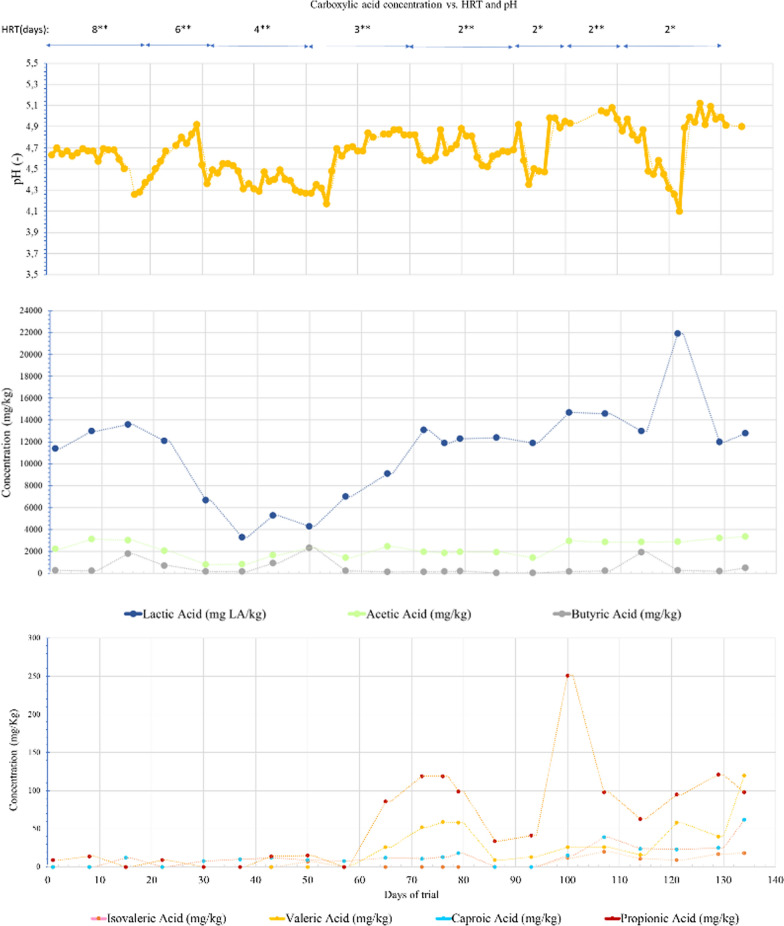
Time-course curves concentration (mg/kg) of lactic acid and the main carboxylic acids generated during OFMSW fermentation for the different feeding strategies Nr.1 to 8 (*: With pH-Adjustment at 7 once a day; **: Without pH-Adjustment), 36 L-digester

The Fig. 1 shows the time-course of VFAs, as well as the concentration of LA, for the different periods in which decreasing HRT is applied as a feeding strategy, to shift the mixed cultures towards lactic fermentation.

After acidification, the total concentration of acids increases, with lactic, acetic, and butyric acids being the predominant onesOver the different feeding strategies (reducing HRTs), the concentration of the lactic acid increased, reaching up to 21,900 mg LA/kg digestate (Fig. 1, Feeding strategy Nr. 8).

Acetic stands out as the most relevant VFA, behind lactic acid, with values between 500–4000 mg/kg being observed. Levels above 2000 mg/kg were observed mainly for lowest HRT. On the other hand, butyric values did not exceed 2000 mg/kg, observing only at three specific moments a certain increase and values around 2000 mg/kg, generally found at levels much lower than acetic.

Regarding the time-course of propionic acid, a certain accumulation in the medium is observed from feeding strategy Nr.4, which remains stable during the experimental trial for a large part of the trial.

Also, in the Fig. 1 it can be observed the time-course of the others carboxylic acids remained below 100 mg/kg, with the accumulation of valeric generally higher than that of caproic, from Feeding strategy Nr. 5 onwards.

In relation to pH, the best accumulations of lactic acid were obtained for 2-day HRT together with pH adjustment around 4.5–5 in the medium (Feeding strategy Nr. 8). However, during the period from day 1 to 90 (without pH adjustment, Feeding strategy Nr. 1–5), this parameter remained between 4.2–4.9, achieving a pH somewhat far from the optimum for LAB (5.5–6) but facilitating lactic fermentation compared to other metabolic pathways, after observing generally higher concentrations of lactic acid compared to other acids analysed in the digestate.

**Fig. 2:**
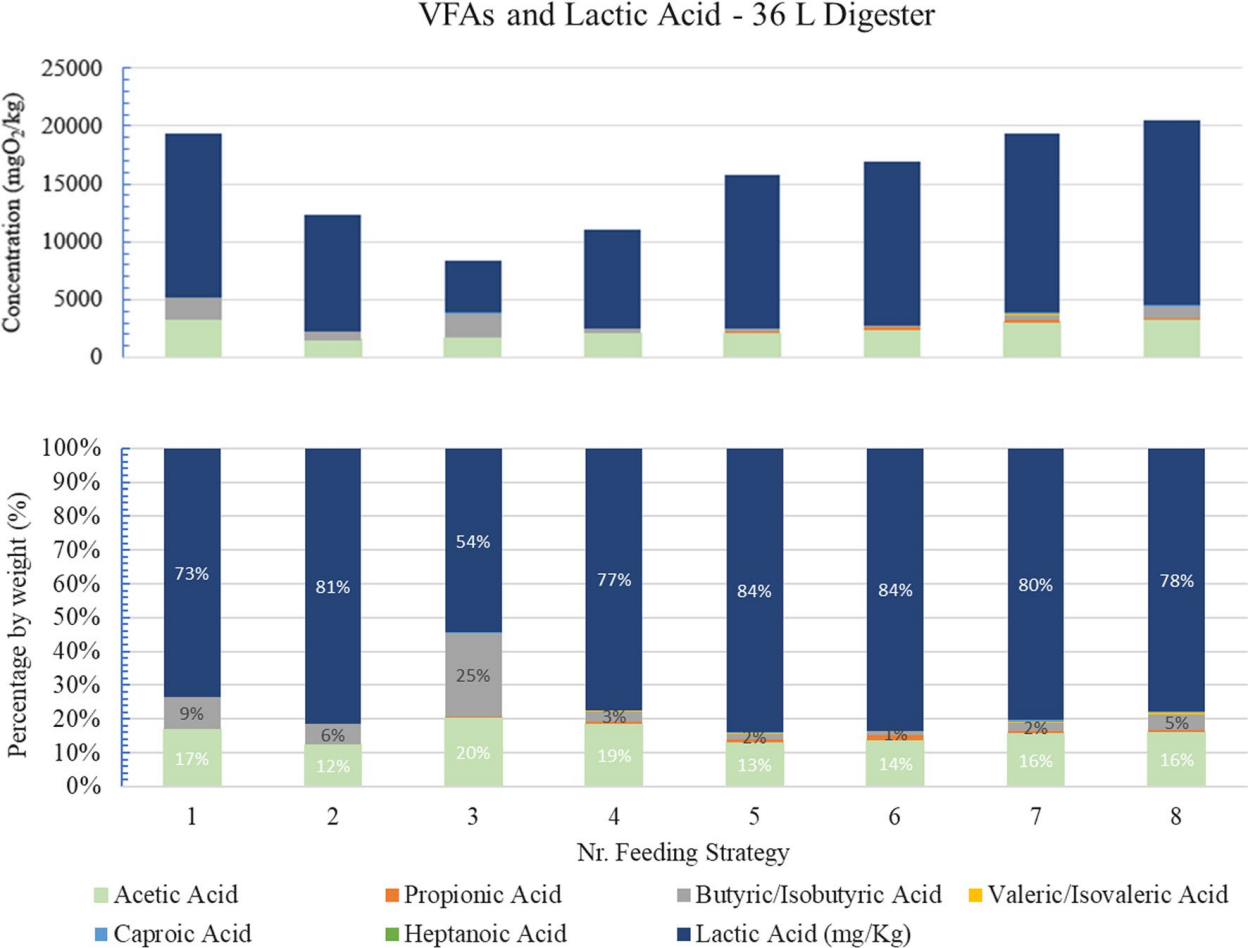
COD concentration and percentage presence by weight of each of the main carboxylic acids generated during OFMSW fermentation under the different operating conditions tested (Feeding strategies Nr. 1–8), 36 L-digester

In relation to the values of the acids in COD concentration, these are summarized in Fig. 2 for comparison.

The presence of lactic acid in relation to all the other acids produced is predominant from the initial days of operation, reaching a maximum (84%) for Feeding Strategy Nr. 5. Highest concentration of main carboxylic acids was achieved in Feeding strategy Nr.8 with a value in terms of COD above 20 gO_2_/kg.

As mentioned above, the results obtained at lab scale on the digestate, analysing total solids, volatile solids, pH, lactic acid, and lactic acid isomers are shown in Table 3.

### Development of lactic acid production on a larger scale: operating conditions and bioaugmentation.

The results included in this section were obtained in the 1500 L-digester, starting from the most favourable operating conditions to produce lactic acid observed at lab scale.

The results obtained at pilot scale on the digestate, analysing total solids, volatile solids, pH, lactic acid, lactic acid isomers and filtered (or soluble) COD are included in Table 4.

**Table 4:** Summary of operating parameters and average results of lactic acid production during OFMSW fermentation trials at pilot scale

Nr. of Feeding strategy	Duration (d)	Operational conditions			Results 1500-L digester (digestate)									Yield (gLA/g TS feeding)
		OLR (kg_VS_/ m^3^d)	OLR (gO_2_/L·d)a	HRT (d)	pH (-)	TS (%)	COD (gO_2_/L)	Lactic acid (g/100 g)	Lactic acid (g/gTS)	Lactic acid (gCOD/kg)	Lactic acid (g_COD_/L)b	% D-Lactic	% L-Lactic	
1	14	11.7	17.5	6	4.10 ± 0.57	5.25 ± 1.77	45.35 ± 8.91	2.00 ± 1.28	0.36 ± 0.12	21.39 ± 13.80	22.24	56.20 ± 4.38	43.80 ± 4.38	0.25
2	12	12.2	18.3	4	4.20 ± 0.14	5.70 ± 0.71	51.13 ± 5.83	2.70 ± 0.00	0.48 ± 0.06	28.69 ± 0.00	29.84	56.60 ± 4.38	43.40 ± 4.38	0.44
3	14	34.3	51.5	2	4.35 ± 0.07	7.30 ± 2.26	45.76 ± 2.95	3.45 ± 0.21	0.50 ± 0.18	37.01 ± 1.69	38.49	52.75 ± 0.92	47.25 ± 0.92	0.38
4	14	25.8	38.7	2^c^	5.00 ± 0.14	7.30 ± 0.28	52.73 ± 1.80	3.65 ± 0.92	0.50 ± 0.15	39.20 ± 9.58	40.77	49.20	50.80	0.54
5	7	26.2	39.4	2^d^	6.35 ± 1.77	6.00 ± 0.14	54.23 ± 8.31	2.15 ± 1.48	0.36 ± 0.26	22.83 ± 16.44	23.74	47.80 ± 3.25	52.20 ± 3.25	0.30
6	7	32.5	48.7	2^e^	5.13 ± 0.06	6.99 ± 0.45	57.55 ± 0.92	3.75 ± 0.02	0.54 ± 0.04	39.95 ± 0.23	41.54	50.35 ± 0.49	49.65 ± 0.49	0.41

*HRT* hydraulic retention time, *OLR* organic loading rate, *TS* total solids, *COD* chemical oxygen demand

^a^Factor of 1,5gCOD per 1 gVS

^b^Average value transformed considering the digestate density (1.04)

^c^pH-Adjustment at 7 once a day

^d^pH-Adjustment at 7 continuously

^e^pH-Adjustment at 7 once a day and addition of 10L of BAL-inoculum

In case of pilot tests, the pH value remained naturally between 4.1–4.4 without pH control in the feeding strategies Nr.1–3.

From feeding strategy 4 onwards, pH in the digester was artificially raised in the media above 5, to approach the optimal pH for the growth of many LAB (5.5–6), resulting in obtaining an average pH value of 5.0–6.4 in feeding strategies Nr.4–6.

At feeding strategies Nr.1–3, the maximum LA production was obtained at the lower HRT tested. At this point, the pH adjustment strategies were applied, observing the highest LA yield in the feeding strategy Nr.6 with addition of 10L-BAL inoculum. The percentage of D-/L isomers in pilot scale trials were between 57–44%, being, in general, slightly higher for D-LA.

**Fig. 3:**
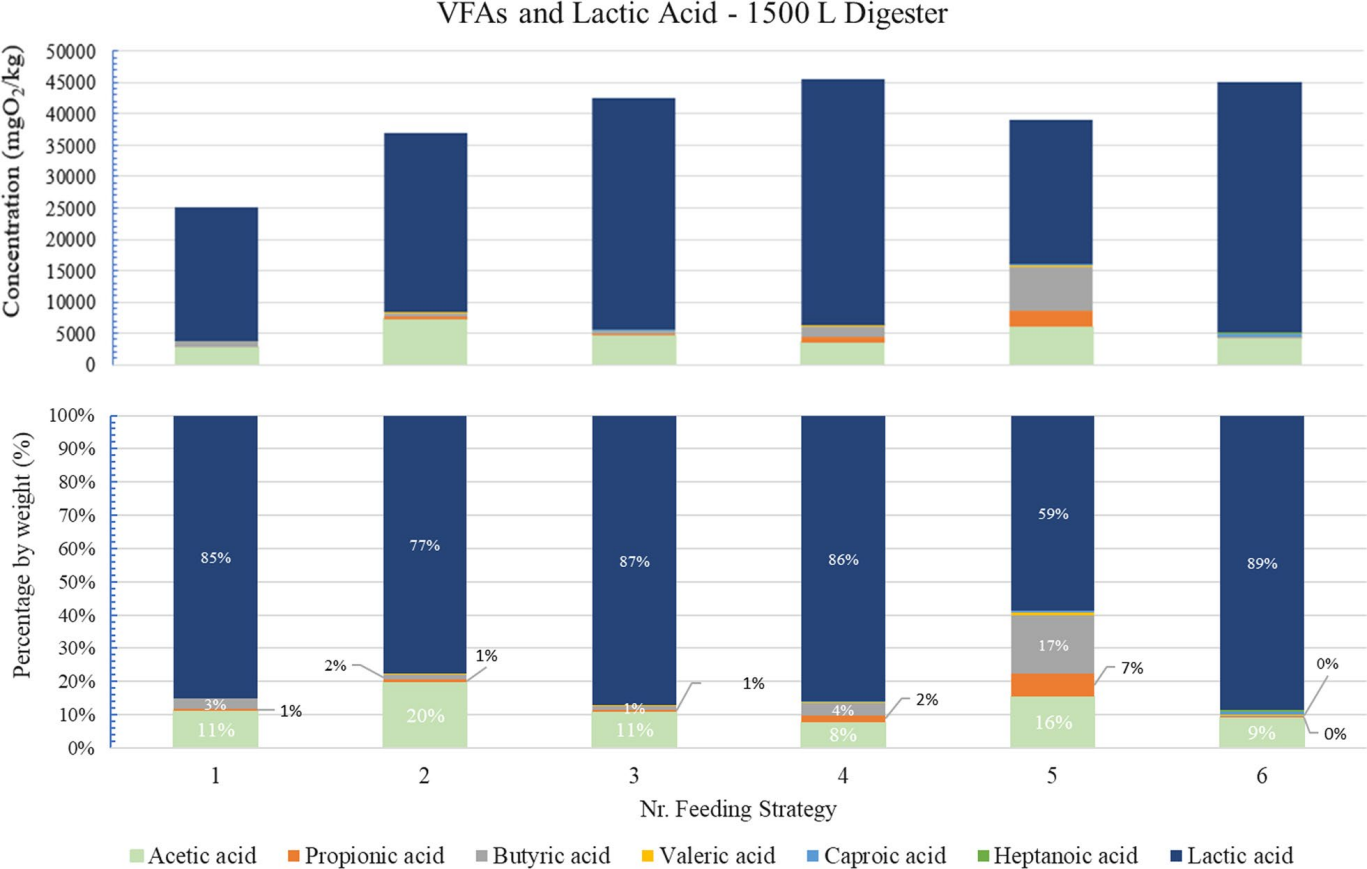
COD concentration and percentage presence by weight of each of the main carboxylic acids generated during OFMSW fermentation under the different operating conditions tested (Feeding strategies Nr. 1–6), 1500 L-digester

Figure 3 summarizes the VFA production in terms of concentration and percentage by weight.

During Feeding Strategy Nr.3, it was possible to see higher lactic acid accumulations compared to the previous HRT, for similar pH values (4.1–4.4).

The presence of lactic acid in relation to all the other acids produced is predominant from the initial days of operation, reaching a maximum for Feeding Strategy Nr. 6 (89%). Highest concentration of main carboxylic acids was achieved in Feeding strategy Nr.4 with a value in terms of COD above 45 gO_2_/kg.

The results obtained in the digestate, in relation to the different main carboxylic acids, are examined in Table 5 for the different Feeding strategies.

**Table 5:** Summary of carboxylic acids production during OFMSW fermentation trials at pilot scale

Nr. of Feeding strategy	Acetic acid	Propionic acid	Butyric acid	Valeric acid	Caproic acid	Heptanoic acid	Lactic acid
1	2794	146	799	18	10	0	21387
2	7279	325	612	76	44	0	28693
3	4638	263	498	65	46	0	37013
4	3581	924	1643	113	85	50	39200
5	6043	2702	6813	291	217	49	22827
6	4129	114	212	57	431	199	39947

After acidification, the total concentration of acids increases, with lactic, acetic, and butyric acids being the predominant ones. From the beginning of the pilot trials, LA was already detected in the digestate at a considerable concentration (21,387 mg_LA_/kg digestate) in comparison with other carboxylic acids (below 3000 mg_LA_/kg digestate). Over the different feeding strategies, the concentration of this acid increased, reaching up to 39,947 mg_LA_/kg digestate (Feeding strategy Nr. 6).

Acetic acid stands out as the most relevant VFAs, behind lactic acid, with values between 2800–6000 mg/kg being observed. Above 3000 mg/kg were achieved from Feeding Strategy Nr.2 onwards. On the other hand, butyric values did not exceed 7000 mg/kg, and generally found at levels much lower than acetic.

Regarding the time-course of propionic acid, a certain accumulation in the medium is observed at Feeding Strategy Nr. 5 (> 2000 mg/kg), which remained below 1000 mg/Kg for a large part of the trial.

Also, in the Table 5, it can be observed the average value for other carboxylic acids that remained below 300 mg/kg, with the accumulation of valeric generally higher than caproic or heptanoic acid.

**Fig. 4:**
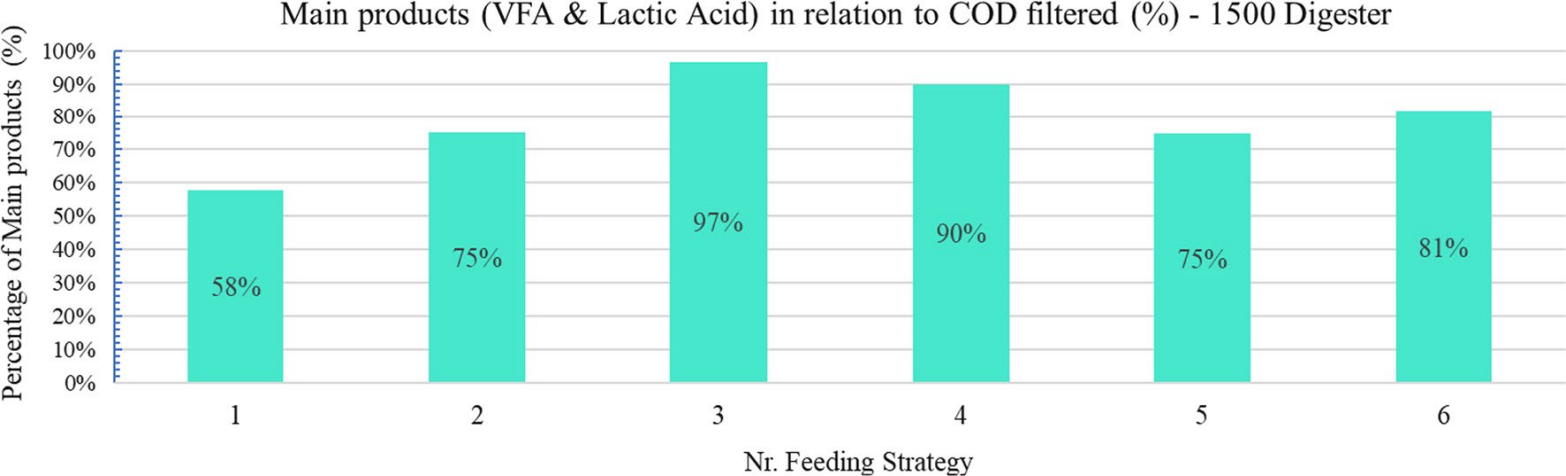
Comparison of main products (VFA and Lactic Acid) related to the COD value filtered from the digestate

Regarding the transformation of the initial organic matter into the target main product (lactic acid), Fig. 4 is presented to analyse the percentage of mentioned products within the solubilized COD (filtered COD).

The presence of lactic acid as a percentage of the total carboxylic acids reached a maximum in Feeding Strategy Nr. 6 (Fig. 3,89%) while total carboxylic acids were more than 80% of the soluble COD of the digestate. However, the highest concentration of main carboxylic acids as part of the total soluble COD of digestate was achieved in Feeding strategy Nr.3 (Fig. 4, 97%).

## Discussion

### Substrate characterization

In the Table 2 it can be observed the results of the characterization of OFMSW used for the experiments at lab and pilot scale. Compared to OFMSW characterization found in literature [38], volatile solids concentration in these samples of OFMSW is analogous and higher than 80%, indicating mentioned substrate has sufficiently high carbon source for LAB growth. Additionally, total solids of OFMSW used in the experiments, before dilution, were slightly higher than in previous research using OFMSW, but being in the range.

Some recent articles report the highest productivity of LA at a C/N ratio of 19:1 ratio and low productivity at the highest C/N ratio of 37:1[31]. The C/N ratio found in the OFMSW used as substrate had an average value of 22:1, so nitrogen supplementation was considered unnecessary in this study.

### Lab-scale experiments: optimisation of pH and HRT

As it can be seen in the Table 3 the pH remained naturally between 4.4–4.7 without pH control in the feeding strategies 1–5. However, it is known that lactic acid bacteria such as lactobacilli is tolerant to extremely low pH condition and can survive at the acidic environment [32–34]. Similarly, to previous studies, the pH adjustment strategies to optimum for lactobacilli increased LA yield [35].

According to the available results in the literature, a 2 days-HRT is estimated to be optimal to maximise lactic acid production in semi-continuous (one feeding per day, pH close to optimum for lactobacilli). In the literature, a new pH control by switching from oscillation to constant pH control has been reported as an efficient method for lactic acid production [9, 25].

In comparison with the results obtained by other authors, it should be noted that the studies found for this type of process were focused on batch processes, but no references were found for continuous processes using real substrates such as OFMSW (Table 1). For comparison with these studies, it is worth mentioning that the lactic acid concentration obtained in this continuous study from OFMSW is in the range of previous research, as it can be observed in Tables 1, 3. Especially the maximum lactic acid productivity point obtained in these trials is around 15–16 gCOD/L, somewhat lower than those obtained in batch with OFMSW as substrate, but relatively close to previous values obtained around 20–28 gCOD/L.

A relevant influence of the pH parameter on lactic acid productivity is observed at lab scale. A pH between 4.5–5 favours the accumulation of lactic acid. In principle, for HRT 2 (d), with a pH closer to 5, the production of lactic acid is favoured. In line with the results obtained, the pH adjustment close to 5 is estimated to optimise the lactic fermentation route compared to others aimed at the production of other carboxylic acids. Low HRT (2 days), pH close to 5 and for applied loads higher than 20 kg_SV_/m^3^·d, the accumulation of lactic acid is favoured, obtaining at laboratory scale results in an order of magnitude, for the analysed substrate, reported in scientific literature (batch feeding) [17, 18, 25]. The percentage of D/L isomers obtained for the most advantageous HRT in terms of lactic acid production is around a racemic mixture of both isomers (50–50%), with the concentration of D-LA being slightly higher than L-LA at the lowest HRT tested, applying a pH adjustment.

In relation to the selectivity in the production of different acids, no generation of valeric acid was observed for HRT 6–8 days. While for HRT 2, 3 and 4, in addition to lactic, acetic, propionic, and butyric acids, some isovaleric/valeric acid production is observed. It is also of interest to link the 2-day HRT with a specific OLR applied. With OLR higher than 20 kg_VS_/m^3^·d, the best accumulations of lactic acid in the medium were obtained.

### Development of lactic acid production on a larger scale: operating conditions and bioaugmentation

In these tests, there is some difficulty in taking an adequate sample in the premix tank, due to the heterogeneity of the sample. In addition, the determination of total solids in the samples also poses some uncertainty due to the heterogeneity of the samples. However, in comparison with lab trials, the possibility of having fresh material of OFMSW daily stands out as very positive.

In addition to that, throughout the experiment, pH values between 4.1 and 5.2 were obtained for the feed mixture, with an average of 4.7 for the mixture before it was introduced into the digester. These values are slightly below the optimum for the growth of LAB, but at the same time allow conditions in the medium in which other microorganisms do not develop or develop only minimally. It is with the feeding strategy of 2 days-HRT that the pH control strategies are applied, introducing NaOH 40% W/W together with the feed up to a value of 7.

Regarding the C/N ratio, the mixture reached values on average around 22 (Table 2). These values are usual in OFMSW, being a very suitable and balanced material for fermentative processes. Subsequently, with daily pH-Adjustment), the lactic acid yields obtained are improved, being preferable to a continuous pH control due to lower production. For this period with daily pH-Adjustment, slightly higher pH values are obtained on average than for the previous feeding strategies.

Finally, in the Feeding strategy Nr. 6, a strategy of bioaugmentation of LAB was applied, adding an inoculum enriched in LAB isolated from the digestate obtained previously. The results of lactic acid accumulation observed are higher than in previous Feeding strategies, being higher than 39,000 mg LA/kg of digestate (Table 4), during more than 7 days of the bioprocess. These results are therefore considered to be sufficiently robust and obtained during more than 3 digester feeding cycles.

The results obtained in the Feeding Strategy Nr. 3, 4 and 6 are remarkable because in these strategies, the soluble COD is mainly composed of VFAs and lactic acid, and it is reasonable to estimate low the content of other compounds or impurities in the solubilised COD. Therefore, for future recovery and purification processes, a positive scenario is proposed, but it must be considered which other compounds are also present, conditioning the subsequent recovery process of VFAs and/or lactic acid.

Finally, it is important to underline that the values obtained for lactic acid in relation to kilograms of digestate (gO_2_/kg) are transformed considering the density of the digestate and expressed as lactic acid in relation to volume of digestate (Table 4). These transformed values allow the comparison with previous results obtained at lab scale, being very positive the values obtained with the experimentation in the prototype, reaching the Feeding strategy Nr. 6, values around 40 gO_2_/L (Table 4), and higher than those found in previous experiments with similar materials (OFMSW, values around 28.4 gO_2_/L, Table 1).

In addition to the high yield values at pilot scale, the high percentage of lactic acid, above 70% (Table 4), for feeding strategy Nr.3, 4 and 6, is noteworthy in terms of soluble COD in the effluent. In general, at the 1500 L-digester, the lower the HRT, the higher the lactic acid accumulation was reached, and, for the lower HRTs studied, higher lactic acid accumulations were achieved than at laboratory scale (> 4 g/100 g at pilot scale; 1.6 g/100 g at laboratory scale, note differences in the degree of freshness of the material) considering both agitation and temperature distribution in said digester to be adequate. Among the possible differences, it is worth mentioning the higher degree of freshness of the prototype feed compared to the pilot scale, which was higher in the case of the prototype, given that the sample was crushed daily and introduced into the digester immediately after crushing. At the pH level achieved with the pH control, a slightly higher pH is observed (5.0) in the 1500 L-digester compared to the laboratory scale trials (4.8), for Feeding periods where the best lactic acid production values are obtained (> 4 g/100 g). In addition to that, the sample used in the pilot trials may contain a higher degree of non-degraded fermentable sugars compared to the sample collected for the laboratory-scale fermenter.

Considering the lactic acid results on a dry basis and the total solids value, the best results were obtained for Feeding Strategy Nr. 6, in which the lactic acid yield in the digestate exceeded the value of 0.5 g LA/gTS (Table 4). In relation to the lactic acid isomers, on average a racemic mixture of both (50–50) was obtained, although certain differences were observed in the different subperiods, with the D-lactic acid isomer being slightly superior to the L-lactic acid isomer (Table 4).

Moreover, regarding the volatile fatty acids generated at the same time during the lactic fermentation process, the accumulation of acetic acid stands out above the rest of the acids, with a certain accumulation of VFAs, to a lesser extent, caproic acids also being observed. In general, in periods with the lowest HRT tested (2 days) and daily pH control, a greater accumulation of lactic acid is observed, as well as a reduction in the rest of the acids observed.

In the Fig. 3 it can be observed the VFAs and lactic acid percentage by weight in digestate. About the accumulation of other VFAs, the increase in butyric acid in the Feeding strategies 1 to 5 is noteworthy, particularly the increase in butyric acid in the medium, after the application of continuous pH control, which favoured the accumulation of the production of this acid, in competition with the bioproduction of lactic acid.

Although in low concentrations, heptanoic acid starts to be present in Feeding strategy Nr. 4 (Table 5), remaining in very low concentrations except during the last week of experimentation, when values of 199 mg_COD_/kg of digestate were observed. Likewise, in the case of caproic acid, a somewhat more relevant presence was also observed during Feeding strategy Nr. 5 (Table 5), as well as during the week of experimentation applying Feeding Strategy Nr. 6 (Table 5).

It should also be noted that the strategy of introducing an inoculum rich in specific LAB was positive, improving the results obtained in the prototype without applying reinoculation. This addition was analysed for one week, with continuous feeding, without observing any biomass washing despite the low operating HRT in this bioprocess, and with an increase in the concentration of lactic acid during the week of applying Feeding Strategy Nr. 6.

In relation to the distribution of isomers in the lactic acid obtained, no major differences are observed for the TRH tested at pilot scale, obtaining a racemic mixture of both isomers (50–50%), and a slight reduction in L-lactic acid as lactic acid accumulation is improved by applying different strategies.

## Conclusions

The results obtained in the 1500 L-digester (pre-industrial scale) confirm that certain operating conditions, which are 2 days-HRT and pH adjusted to around 5.0 on a daily basis, favour the production of lactic acid from mixed cultures. For said conditions, and by introducing LAB-rich inoculum developed from the separation of LAB in the effluent of the prototype, lactic acid accumulations were achieved up to values of 41.5 g_O2_/L (higher than those reported in the literature of around 28 g_O2_/L). These values of lactic acid accumulation obtained, expressed as COD, reached more than 70% of the filtered COD measured in the digestate.

To conclude, these results are an important advance with respect to previous studies since the system is analysed in continuous operation (Working volume 1500 L) using fresh OFMSW and lactic acid production is maintained during sufficient operating HRTs. However, there is still room for improvement of the bioprocess (digester configuration, bioaugmentation strategies, etc.), for further increasing lactic acid accumulation. This is relevant because the subsequent recovery processes of the by-products obtained are highly conditioned by the concentration reached and impurities present.

## Methods

### Substrate and inoculum

The OFMSW used as substrate was collected from the solid urban waste treatment plant in Algimia de Alfara (Valencia), specifically organic fraction from selective collection (Additional file 1: Figure S1). Foreign materials were manually removed, and the clean OFMSW was then pre-shredded in a Thermomix^®^ and stored frozen until use (− 18 °C) for lab scale trials (See Additional file 2: Figure S1)

Prior to being fed to the digesters, OFMSW samples were milled and diluted with tap water to reduce the total solids content of the feed to 7.6–12.5% (Table 2), to facilitate the operation in a continuous stirred tank reactor (CSTR), and considering previous start-up tests that set a solids content of less than 15% as the operating limit to minimize possible mechanical failures of the agitation system.

The starter inoculum for the anaerobic digestion was collected from a wastewater treatment plant (WWTP) in Paterna (Valencia, Spain), from a mesophilic anaerobic digester fed with sewage sludge.

Similarly to lab scale trials the OFMSW used as substrate for pilot trials was collected from the solid urban waste treatment plant in Algimia de Alfara. OFMSW was shredded for pilot trials using a grinding machine prior to be used in the 1500 L-digester.

Approximately 437 L of inoculum and 437 L of water were used for inoculation in pilot trials (1500 L scale), with the digester temperature set at 55 °C.

Considering the presence of indigenous microorganisms (*Lactobacillus*) in the substrate itself and benefits of inoculum from non-sterilized environments, it was decided in both lab and pilot trials to start with mentioned inoculum from an anaerobic digester, which was acidified by overload to favour suitable conditions for lactic acid fermentation (pH), and then, start the feeding with OFMSW (Table 3, 4).

### Experimental procedure and set-up

The experimental design consisted in different feeding strategies in which the process parameters influencing LA yield (temperature, pH, HRT) [18, 24, 31] were studied. The feeding cycle consists of 7 days of feeding for lab trials and 5 days of feeding, with a double load being introduced on the fifth day for pilot trials. In the last ones, during the weekend the digester was not fed.

Additionally, a novel bioaugmentation strategy was applied in the pilot trials, for the best HRT and pH conditions identified at lab scale.

Temperature was kept at thermophilic range (50–55 °C), since it has been reported as optimum for LA production and selectivity over total products of fermentation [5, 24, 26]. As an additional benefit, lower concentration of pathogens in the effluent of the process is expected with thermophilic range.

The experiments were carried out in lab and pilot anaerobic digesters.

The lab digester (36L total volume, 30L working volume) has a CSTR configuration with mechanical agitation that works intermittently every 5 min.

At lab digester, a strategy of progressive HRT reduction was chosen (Table 3), aimed to reach the optimal HRT to maximise LA yield in a stable acidogenic process. Then, HRT was kept in its optimal value and two pH strategies were applied to evaluate possible benefits for LA production: (i) daily pH adjustment by a dilution of 40% NaOH, feeding strategy 6; (ii) no pH control applied feeding strategy 7 and (iii) daily pH adjustment was applied again, feeding strategy 8, for 4 HRTs and re-feeding for 5 HRTs.

The digestate was analysed daily for pH and IA/PA ratio, while total solids, volatile solids, VFAs concentration and lactic acid were measured on a weekly basis. The percentage of each lactic acid isomer was measured for the best conditions in terms of lactic acid concentration obtained in the trials.

The pilot digester (1500L total volume, 1000L working volume) was in Algimia de Alfara (Valencia). It has a CSTR configuration with mechanical agitation that works continuously. The first feeding strategies aimed to validate and reach the optimal operating conditions of pH and HRT found in the lab trials. Then, a novel bioaugmentation strategy (see description in the next section) was applied. Details of the specific operating conditions applied in each feeding strategy at pilot scale are presented in Table 4.

Total solids and volatile solids were analysed lab and pilot scale trials in the OFMSW feeding, and C/N ration only in pilot scale trials. The digestate was analysed in lab and pilot scale trials for total solids, volatile solids, lactic acid, D- and L-lactic acid, pH and VFA. In addition to that, filtered COD was measured in pilot scale trails.

### Bioaugmentation procedure

It was developed an inoculum by selecting LAB from 1500 L-digester digestate. In this work, a selection procedure of LAB from the digesters used was carried out. For this purpose, a 20 ml volume of digestate was centrifuged at 13,000 rpm for 10 min. After that, 1 g of the pellet was used to inoculate closed bottles with 83.33 ml of MRS culture medium (SIGMA). MRS culture medium is a selective medium for LAB with high efficiency [11]. The inoculum cultures were maintained at 42 °C and 200 rpm during 24 h in an orbital shaker.

250 ml of inoculum were used to inoculate 4000 ml of MRS-broth in a stirred tank bioreactor (5 L of total volume). The conditions of the bioreactor were: 300 rpm, 42 °C temperature (optimum LAB), pH control at 5.8 (optimum LAB) with a 10 M NaOH solution. After 24 h, the final volume is kept under refrigeration.

With a view to inoculate a 1500 L-digester, about 10 L (1% v/v) of LAB-inocula were produced in the conditions previously mentioned.

### Analytical methods

TS and VS were analysed by gravimetry, according to standard method 2540-B and 2540-E, respectively [37]. The COD was determined using the WTW™ 252,070 spectrophotometric kit. The concentration of the different volatile fatty acids (VFA) acetate, propionate, iso-butyrate, n-butyrate, iso-valerate, n-valerate, iso-caproate, caproate and heptanoate were analysed using a thermo gas chromatograph with an HP-FFAP capillary column (Agilent, US) and a flame ionization detector (FID). LA was determined using a waters High Performance Liquid Chromatograph with a photodiode array detector (LC-PDA) while the measurement of the percentage of each enantiomer of lactic acid was measured by Assay Enzymatic Kit (D-/L-Lactate) from Megazyme. The IA/PA ratio was measured based on a titration workstation to determine the ratio of the acid concentration and the compensating capacity of the fermentation substrate. This IA/PA ratio was determined using an automatic titrator model TIM 840 Potentiometric Titration Manager monoburette.

## Supplementary Information

**Additional file 1**: **Figure S1**. Image of the residue used as a substrate in this work, OFMSW. **Figure S2**. Picture of the waste shredding system used in this work.

## Data Availability

All data generated or analyzed during this study are included in this published article.

## Abbreviations

OFMSW: Organic fraction of municipal solid waste
LA: Lactic acid
COD: Chemical oxygen demand
OLR: Organic loading rate
HRT: Hydraulic retention time
LAB: Lactic-acid bacteria
VFAs: Volatile fatty acids
CAGR: Compound annual growth rate
PLA: Polylactic acid
CSTR: Continuous stirred tank reactor
WWTP: Wastewater treatment plant
IA/PA: Ratio of intermediate alkalinity or volatile organic acids (IA) over the partial alkalinity or alkaline buffer capacity (PA)
STBR: Stirred tank bioreactor
TS: Total solids
VS: Volatile solids
COD: Chemical oxygen demand
