# Supplementary material for: New efficient meta-fermentation process for lactic acid production from municipal solid waste
Source: Microb Cell Fact. 2022 Nov 5;21:233. doi: 10.1186/s12934-022-01960-9 (PMC9636838; doi:10.1186/s12934-022-01960-9)
Supplement: Supplementary file 1 — Additional file 1: Figure S1. Image of the residue used as a substrate in this work, OFMSW. Figure S2. Picture of the waste shredding system used in this work. [file 12934_2022_1960_MOESM1_ESM.docx]

Additional file

Figures

Figure S1. Image of the residue used as a substrate in this work, OFMSW.

Figure S2. Picture of the waste shredding system used in this work.


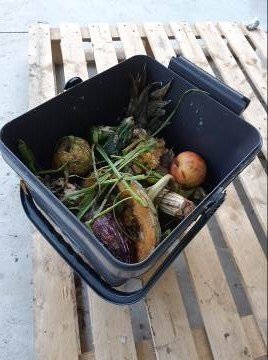


Figure S1. Image of the residue used as a substrate in this work, OFMSW.


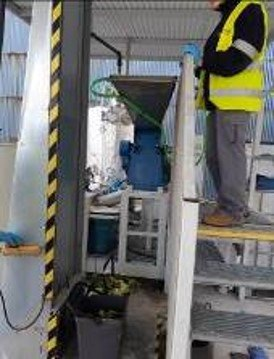


Figure S2. Picture of the waste shredding system used in this work.
